# Identification of the Mutational Landscape of Gynecological Malignancies

**DOI:** 10.7150/jca.46174

**Published:** 2020-06-08

**Authors:** Suresh Chava, Romi Gupta

**Affiliations:** Department of Biochemistry and Molecular Genetics, University of Alabama at Birmingham, Birmingham, AL, 35233, USA.

**Keywords:** gynecological malignancies, TCGA, Oncomine, biomarker, therapeutic

## Abstract

**Background:** Cancer is a complex disease that arises from the accumulation of multiple genetic and non-genetic changes. Advances in sequencing technologies have allowed unbiased and global analysis of patient-derived tumor samples and the discovery of genetic and transcriptional changes in key genes and oncogenic pathways. That in turn has facilitated a better understanding of the underlying causes of cancer initiation and progression, resulting in new therapeutic targets.

**Methods:** In our study, we have analyzed the mutational landscape of gynecological malignancies using datasets from The Cancer Genome Atlas (TCGA). We have also analyzed Oncomine datasets to establish the impact of their alteration on disease recurrence and survival of patients.

**Results:** In this study, we analyzed a series of different gynecological malignancies for commonly occurring genetic and non-genetic alterations. These studies show that white women have higher incidence of gynecological malignancies. Furthermore, our study identified 16 genes that are altered at a frequency >10% among all of the gynecological malignancies and tumor suppressor TP53 is the most altered gene in these malignancies (>50% of the cases). The top 16 genes fall into the categories of either tumor suppressor or oncogenes and a subset of these genes are associated with poor prognosis, some affecting recurrence and survival of ovarian cancer patients.

**Conclusion:** In sum, our study identified 16 major genes that are broadly mutated in a large majority of gynecological malignancies and in some cases predict survival and recurrence in patients with gynecological malignancies. We predict that the functional studies will determine their relative role in the initiation and progression of gynecological malignancies and also establish if some of them represents drug targets for anti-cancer therapy.

## Introduction

Cancer arises from the accumulation of multiple genetic and non-genetic alterations that drive cancer-cell growth and progression 1-3. Advances in large-scale sequencing technologies have allowed sequencing of thousands of tumors and matched normal tissues from patients with different types of cancer and led to the discovery of key alterations that drive cancer development 4. The Cancer Genome Atlas (TCGA) contains molecular characterizations of more than 20,000 primary tumors and matched normal tissues spanning 33 cancer types 5, 6. TCGA datasets can be used to identify genetic and non-genetic alterations for subsequent functional validation and drug discovery.

Gynecological malignancies are a leading cause of cancer-related death in women 7. The most common gynecologic malignancies that affect human female reproductive organs are cervical cancer, uterine cancer, ovarian/fallopian tube cancer, vaginal cancer, and vulvar cancer 8. In 2018 in the United States alone, approximately 22,240 patients were newly diagnosed with ovarian cancer, of which around 14,070 were expected to die of the disease 9. Cervical cancer is the second most common cancer in women worldwide and has an especially high incidence in developing countries, which account for almost 85% of diagnosed cases 10. The treatment and cure of gynecological malignancies remains a challenge, because many cases are not diagnosed until the disease has reached an advanced stage 11. Detailed studies are needed to identify early biomarkers that can predict, diagnose, and monitor gynecological cancers.

Here, we analyzed gene mutation and copy number alterations using TCGA data from 607 patients with cervical cancer 5, 12 1,672 cases patients with ovary/fallopian tube cancer 5, 12-14 1,799 patients with uterine cancer 12, 15-18 and 15 patients with vulvar/vaginal cancer 19. Overall, our analyses identified a set of genes that are frequently altered by mutations or copy-number alterations in gynecological malignancies. Some of those genetic alterations were specific to gynecological malignancies, whereas others were common to other cancer types, such as KRAS and TP53 mutations. Several of the genes that were specifically altered in gynecological malignancies were previously shown to regulate transcription or signaling thus affecting tumor growth, metastasis, treatment outcome, and patient survival 20, 21. In summary, we identified several novel alterations in gynecological malignancies that should be investigated using functional validation assay to establish their role in driving gynecological malignancies and as potential drug targets.

## Results

### Evaluation of cancer type, racial/ethnic disparity, and diagnosis age in gynecological malignancies

**Figure 1:**
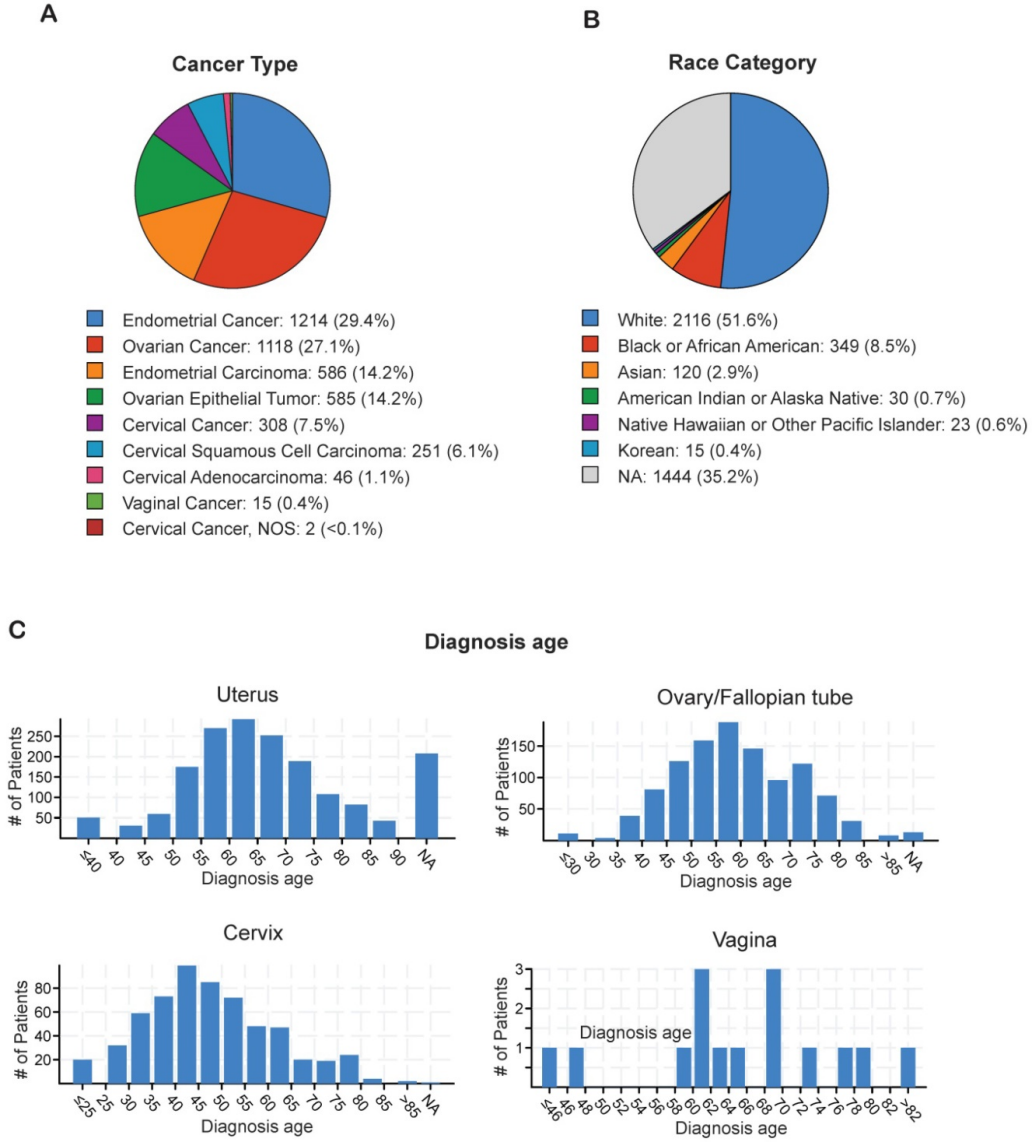
** Analysis of ethnicity and diagnosis age of patients with gynecological malignancies** (**A**) Percentage of each of type of gynecological malignancy in the TCGA dataset from cBioportal. (**B**) Percentage of each race/ethnicity among patients with any gynecological malignancy in the TCGA dataset from cBioportal. (**C**) Diagnosis age of patients with different kinds of gynecological malignancies in the TCGA dataset from cBioportal. NA shown in panel B and C stands for not applicable.

In order to identify the most prevalent types of gynecological malignancies (cervical, uterine, ovarian/fallopian tube, vaginal, and vulvar), we analyzed individual TCGA datasets for cervical cancer (n=607), ovarian/fallopian tube cancer (n=1,672), uterine cancer (n=1,799), and vulvar/vaginal cancer (n=15) using cBioPortal (**Table 1**). The majority of the gynecological malignancies were attributed to endometrial cancer (29.4%) and ovarian cancer (27.1%), whereas cervical cancer (7.5%) and vaginal cancer (0.1%) occurred at lower frequencies (**Figure 1A**). The incidence of gynecological malignancies was highest among White women (51.6%), followed by Black or African American women (8.5%), Asian women (2.9%), Native American women (0.7%), Korean women (0.4%), and Native Hawaiian or other Pacific Islander women (0.6%; **Figure 1B**). Another important factor that affects tumor development and treatment outcomes is the age at diagnosis 22. Therefore, we analyzed the age at diagnosis among the patients with each type of gynecological malignancy. Although the majority of cervical cancer cases were diagnosed early, when the patients were between 40 and 45 years of age, both endometrial cancer and ovarian cancer were diagnosed at a later age, when the patients were between 55 and 65 years of age (**Figure 1C**). These results indicate that endometrial and ovarian are the leading gynecological malignancies with incidence being highest in white women between 55 and 65 years of age.

### Mutational load and copy-number alterations in gynecological malignancies

**Figure 2:**
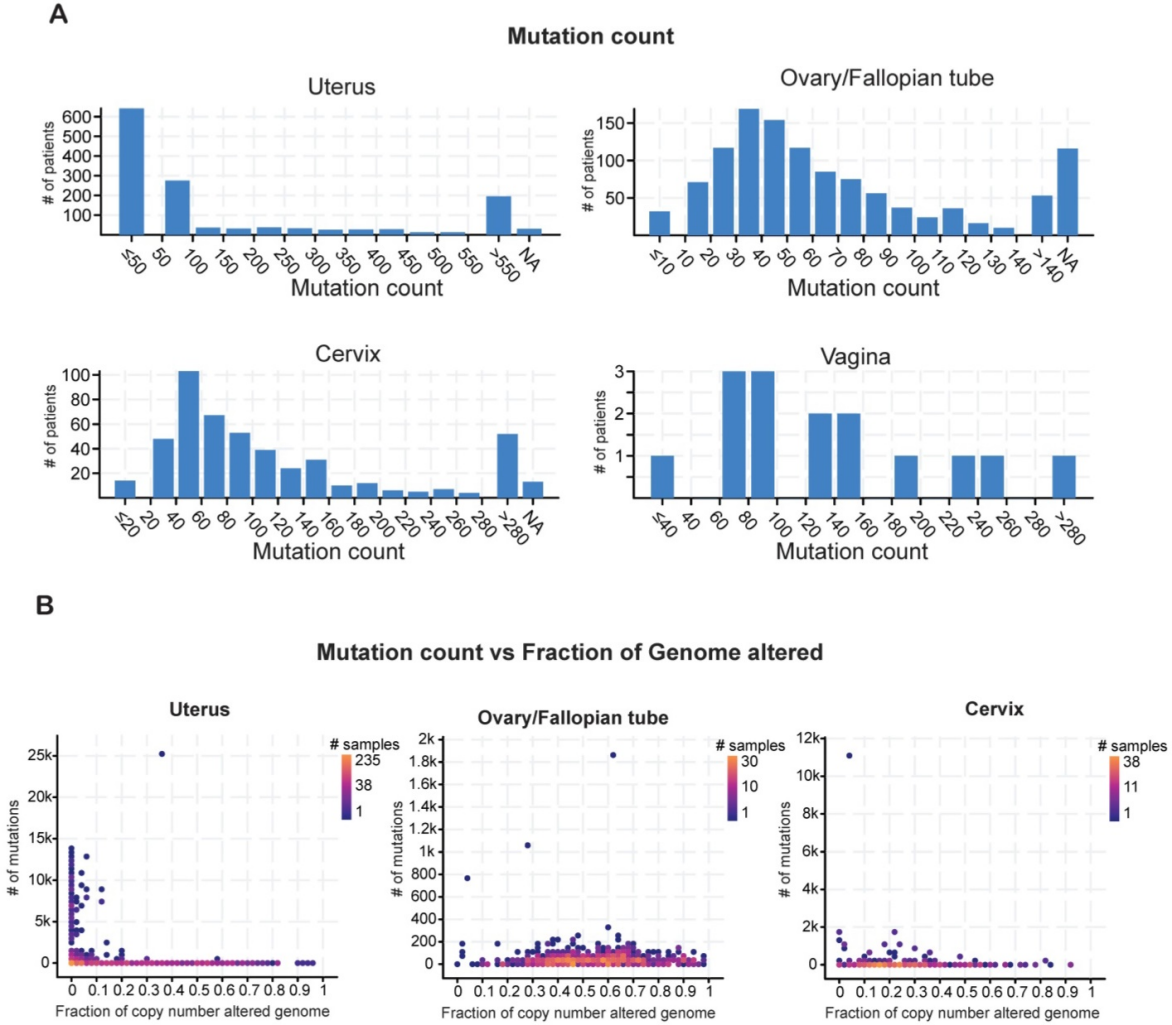
** Analysis of mutation counts in gynecological malignancies** (**A**) Mutation counts in patients with different kinds of gynecological malignancies in the TCGA dataset from cBioportal. (**B**) Mutation counts versus fraction of genomes altered by copy-number changes for different kinds of gynecological malignancies in the TCGA dataset from cBioportal. NA shown in panel A stands for not applicable.

Cancer cell genomes have a higher global mutational burden than normal, healthy cell genomes 23. Cancer-specific mutations play a key role in prognosis and can be used as biomarkers to predict patient responses to immunotherapy or chemoradiation therapy 24. Therefore, we evaluated the mutational burden in each type of gynecological malignancy by counting the mutations in each tumor sample. Most of the samples of uterine cancer harbored fewer than 50 mutations. Most of the ovarian tumors had a mutational burden of 30-40 alterations, whereas the cervical tumors had higher mutational burdens in the range of 40-60 alterations (**Figure 2A**).

The mutational burden comprises missense, truncating, in-frame, and other mutations as well as copy-number alterations, including loss of heterozygosity 25. To determine how much of the mutational burden was due to copy-number alterations, we plotted the number of mutations against the fraction of cancer genomes with copy-number alterations for each cancer type. Patients with uterine cancer had relatively high numbers of mutations but comparatively few copy-number alterations (**Figure 2B**). By contrast, patients with ovarian cancer had fewer mutations but a higher incidence of copy-number alterations. In cervical cancer, the numbers of mutations and copy-number alterations were both low (**Figure 2B**). These results suggest that these gynecological malignancies differ from each other in terms of having different mutational burden and also having different kind of gene alterations.

### Frequently altered genes in gynecological malignancies

**Figure 3:**
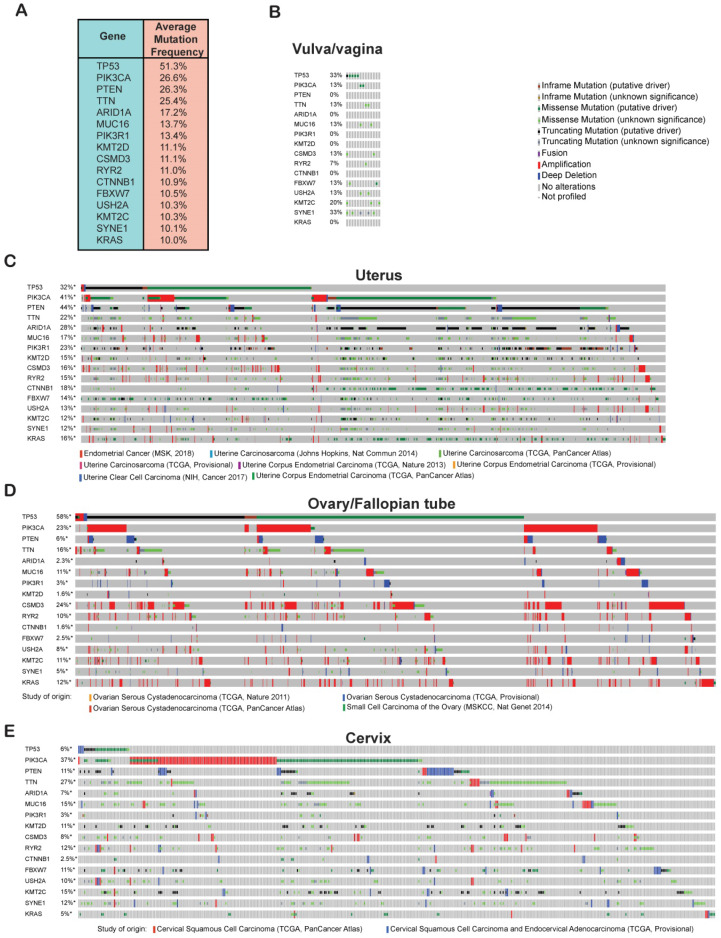
** Frequently altered genes in gynecological malignancies.** (**A**) Genetic alterations (somatic mutations and copy-number changes) were analyzed in different kinds of gynecological malignancies in the TCGA dataset from cBioportal. The table shows the genes that are commonly altered at a frequency >10% among all of the gynecological malignancies. (**B-E**) Missense, in-frame, truncating, amplification, deletion, and fusion mutations were analyzed in the genes that were altered at a frequency >10% in panel a. Missense, in-frame, truncating, amplification, deletion, and fusion mutations are shown separately for (**B**) vulvar/vaginal cancer, (**C**) uterine cancer, (**D**) ovarian/fallopian tube cancer, and (**E**) cervical cancer in different TCGA datasets in cBioportal.

Cancer development and progression occur as a result of the accumulation of genetic and epigenetic alterations in a large number of genes 26. In order to identify genes that are commonly altered in gynecological malignancies, we analyzed the TCGA data for mutations and copy-number alterations in all of the cervical, uterine, ovarian/fallopian tube, vaginal, and vulvar cancer samples using cBioportal. We found that 51% of the gynecological malignancies had alterations in the tumor protein 53 (*TP53*) gene and was the number one mutated gene and therefore probably an important genetic factor driving the growth and proliferation of gynecological malignancies (**Figure 3A**). Other than *TP53*, we found that phosphatidylinositol-4,5-bisphosphate 3-kinase, catalytic subunit alpha (*PIK3CA*) and phosphatase and tensin homolog (*PTEN*) were also highly mutated, each with a frequency of 26% among all of the samples. Mutational activation of the oncogene *PIK3CA* and loss of the tumor-suppressor gene *PTEN* can activate AKT signaling, which plays an important role in cancer development 27, 28. We also found the titin (*TTN*) gene to be altered at nearly the same frequency as *PIK3CA* and *PTEN* among all the gynecological malignancies. Missense mutations in *TTN* has been reported in lung squamous cell carcinoma and has been shown to promote their growth 29. Other genes that were mutated at frequencies of 10% or higher among the gynecological cancer samples were AT-rich interactive domain-containing protein 1A (*ARID1A*), mucin 16 (*MUC16*), phosphatidylinositol 3-kinase regulatory subunit alpha (*PIK3R1*), histone-lysine N-methyltransferase 2D (*KMT2D*), CUB and sushi multiple domains 3 (*CSMD3*), ryanodine receptor 2 (*RYR2*), catenin beta-1 (*CTNNB1*), F-box/WD repeat-containing protein 7 (*FBXW7*), usherin (*USH2A*), lysine N-methyltransferase 2C (*KMT2C*), spectrin repeat-containing nuclear envelope protein 1 (*SYNE1*), and KRAS proto-oncogene (*KRAS*) (**Figure 3A**). Based on the previous studies, which have shown that many of these genes are involved in various processes of growth and progression in other cancer type, it is likely that these candidate genes that are mutated at frequencies >10% will alter signaling pathways or other cellular processes to promote the growth and proliferation of gynecological malignancies too.

After identifying these 16 commonly altered candidate genes in gynecological malignancies, we next wanted to know their mutational status in each type of gynecological malignancies. To do so, we analyzed TCGA dataset encompassing 607 patients with cervical cancer 5, 12 1,672 cases patients with ovary/fallopian tube cancer 5, 12-14 1,799 patients with uterine cancer 12, 15-18 and 15 patients with vulvar/vaginal cancer 19 and plotted the mutations frequencies for each of the 16 identified genes separately for each type of gynecological malignancies. As shown in **Figure 3B-E**, we found that each of the 16 genes were highly mutated in various TCGA datasets across different gynecological malignancies types emphasizing on the important role that they might play in the development of each of these gynecological malignancies. In sum, our analysis identified 16 most important genes that are altered with >10% frequency across different gynecological malignancies types.

### Mutational status of frequently altered genes in gynecological malignancies

**Figure 4:**
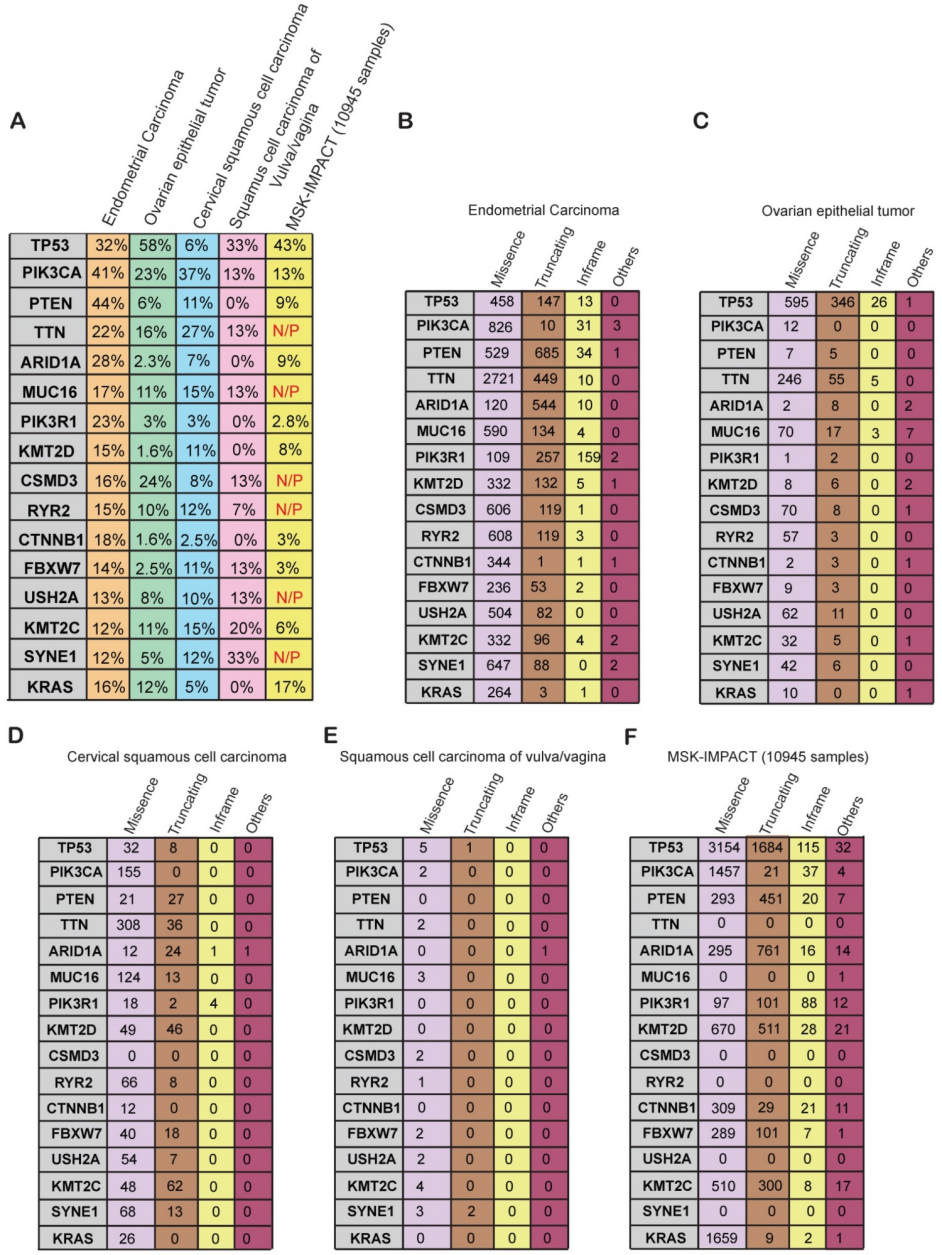
** Specific genes and their alteration status in gynecological malignancies** (**A**) Percentage alteration (mutation and copy-number changes) in the genes mutated at >10% frequency in different kinds of gynecological malignancies and in the MSK-IMPACT cohort containing 10,945 samples of different kinds of cancer. (**B-F**) Number of missense, truncating, in-frame, and other mutations present in different kinds of gynecological malignancies and the MSK-IMPACT cohort.

We next asked whether the genes that were altered at frequencies of 10% or greater in gynecological malignancies are altered specifically in gynecological malignancies or in a more widespread manner across other cancer types. To know that we compared the mutational frequency of each of the 16 genes in the various gynecological malignancies with that in other cancer types present in the MSK-IMPACT cohort. The MSK-IMPACT cohort contains 10,945 samples that encompass 62 principal tumor types including colorectal cancer, prostate cancer, glioma, pancreatic cancer, bladder cancer, melanoma, renal cell carcinoma, breast carcinoma, non-small-cell lung cancer, head and neck carcinoma, and other types of cancers 30. We found that *PIK3CA, PTEN, ARID1A, PIK3R1, KMT2D, CTNNB1, FBXW7, KMT2C* were mutated at a much higher frequency in gynecological malignancies than in other cancer types (**Figure 4A**). Furthermore, *TTN, MUC16, CSDM3, RYR2, USH2A*, and *SYNE1* were frequently mutated in gynecological cancers but not in the MSK-IMPACT samples (**Figure 4A**), suggesting that they play specific roles in the development of gynecological malignancies. Those genes should be analyzed further to uncover their specific role in gynecological malignancies and potentially identify new opportunities for therapy.

Next, to know what kind of somatic mutations were present in these 16 genes, we determined the frequencies of missense, truncating, in-frame, and other type of mutations in each gene. Uterine carcinoma had high numbers of missense and truncating mutations in almost all of the frequently mutated genes (**Figure 4B**). In ovarian cancer, most of the mutations were missense mutations except for the mutations in *TP53* (**Figure 4C**). We found that *TP53* gene encompasses comparatively higher number of truncating mutations apart from missense mutation as compared to other genes in various gynecological malignancies (**Figure 4C**). It has been shown that approximately 80% of *TP53* gene mutations are missense mutations that result in single amino acid substitutions in the p53 protein. Missense mutations in *TP53* leads to accumulation of the mutant protein in the cells because of delayed or impaired degradation, which is then detected as strong and diffuse nuclear accumulation of p53 protein. Additionally, it's been reported that most of the remaining *TP53* mutations are frameshift or nonsense that leads to either absence or truncated versions of the *TP53* protein. The expression of p53 protein in tumors expressing these frameshift or nonsense *TP53* mutations is not detectable 31. Similarly, missense mutations were more common than other types of mutations in cervical cancer and vulvar/vaginal cancer (**Figure 4D-E**). In the MSK-IMPACT cohort, the majority of the mutations were missense mutations, but there were truncating and in-frame mutations present as well in several of the genes (**Figure 4F**). These results indicate that the majority of the somatic mutations found in these genes are of missense kind followed by truncating mutations. These mutations either effect the activation of protein or lead to truncated protein.

### Genes that are overexpressed or repressed in ovarian cancer

**Figure 5:**
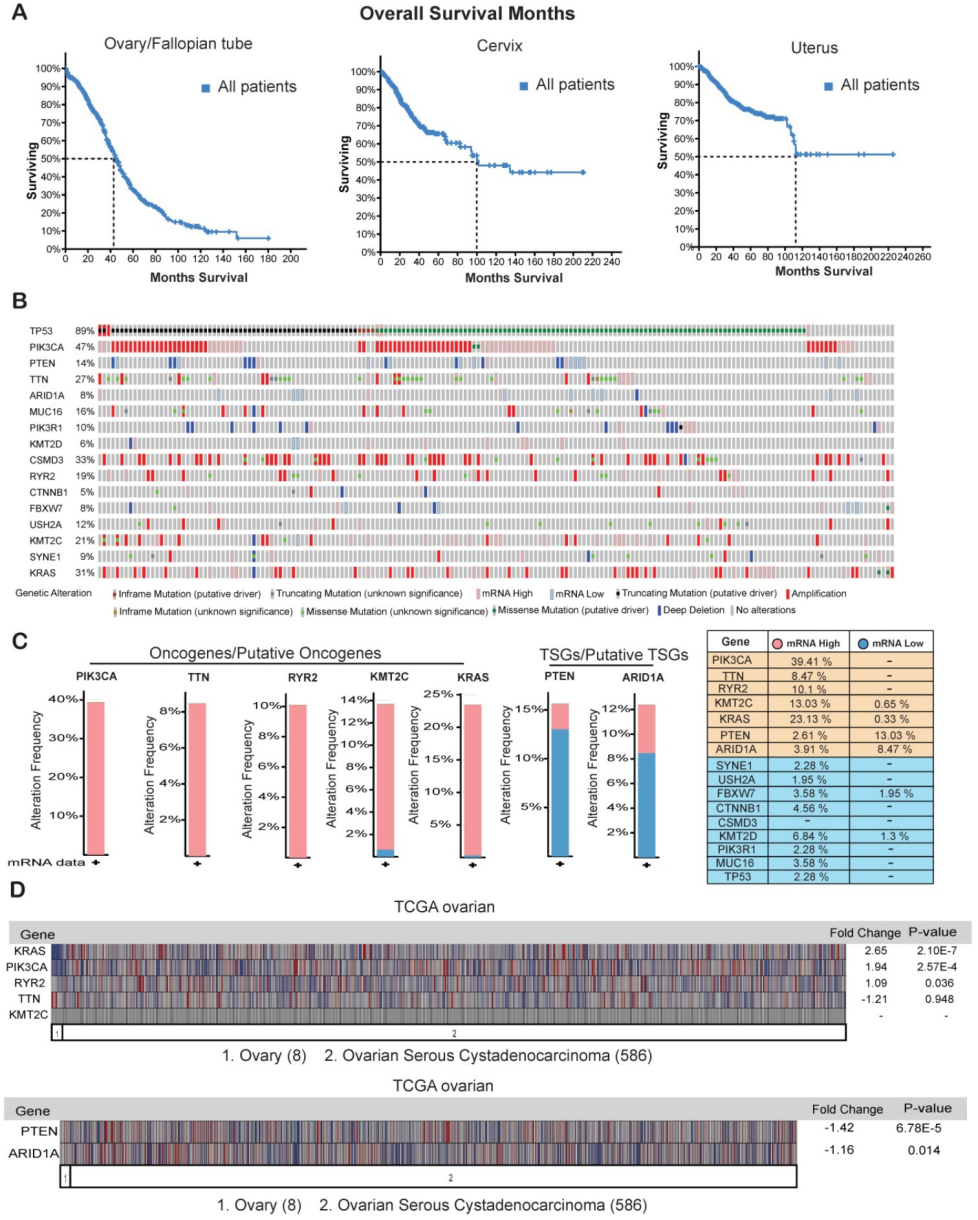
** Genes that are overexpressed or repressed in ovarian cancer.** (**A**) Overall survival in months of patients with ovarian/fallopian tube cancer, cervical cancer, and uterine cancer in the TCGA dataset from cBioportal. (**B**) Missense, in-frame, truncating, amplification, deletion, and fusion mutations, putative copy-number alterations from GISTIC algorithms and mRNA expression (upregulation and downregulation) of the shown genes are analyzed using ovarian serous cystadenocarcinoma (TCGA, Provisional; 606 samples) datasets. (**C**) Bar graph for mRNA expression of the genes that were either highly upregulated or downregulated along with mRNA expression data for all the genes in samples from ovarian serous cystadenocarcinoma (TCGA, Provisional; 606 samples) datasets is shown. (**D**) mRNA expression of the selected genes analyzed using the Oncomine TCGA dataset. Fold-change values and their significance are shown.

Ovarian cancer has been shown to be the most lethal type of gynecological malignancy 32. We found that although half of the patients with cervical cancer or uterine cancer survived for at least 100 months, half of the ovarian cancer patients died within the period of 40 months (**Figure 5A**) clearly indicating that ovarian cancer has the highest rates of early mortality and cancer-related death among the gynecological malignancies. The overall 5-year survival rate among patients with epithelial ovarian cancer is approximately 30% and has not improved over the last three decades because of a lack of effective screening for early-stage disease 33, 34.

Our goal is to identify appropriate genetic biomarkers for ovarian cancer that will allow early screening in patients before any signs or symptoms of disease develop. Additionally, genetic testing of such biomarkers would help to identify hereditary risk of ovarian cancer, especially in families with a strong history and prevalence of ovarian cancer 35-37. We examined the mRNA expression profiles of the 16 genes that were frequently mutated in the ovarian cancer samples from the TCGA provisional dataset. Of the 16 genes that were frequently mutated or had copy-number changes in the ovarian cancer samples (**Figure 4A and 4C**), 5 were transcriptionally overexpressed in the cancer tissues (*PIK3CA* (39.41%), *TTN* (8.47%), *RYR2* (10.1%), *KMT2C* (13.03%), and *KRAS* (23.13%)), and 2 were strongly repressed at the mRNA level (*PTEN* (13.03%) and *ARID1A* (8.47%); **Figure 5B** and **5C**). These results suggest that apart from harboring somatic mutations and copy-number changes, some of these genes have altered mRNA expression levels and probably corresponding protein levels also. We also checked the expression levels of these seven genes in another mRNA expression dataset called TCGA oncomine. TCGA oncomine contains eight normal ovary samples and 586 ovarian serous cystadenocarcinoma samples. The results obtained from TCGA oncomine were similar to those that we obtained from the TCGA genomic dataset from cBioPortal namely *KRAS* and *PIK3CA* mRNA expression was significantly upregulated, and *PTEN* mRNA expression was significantly downregulated (**Figure 5D**) in ovarian serous cystadenocarcinoma samples as compared to normal ovary samples**.** These results validate that many of the genes that are somatically mutated in ovarian cancer patient samples are also altered at mRNA and proteins levels.

### Effect of the overexpressed or repressed genes on the survival of patients with ovarian cancer

**Figure 6:**
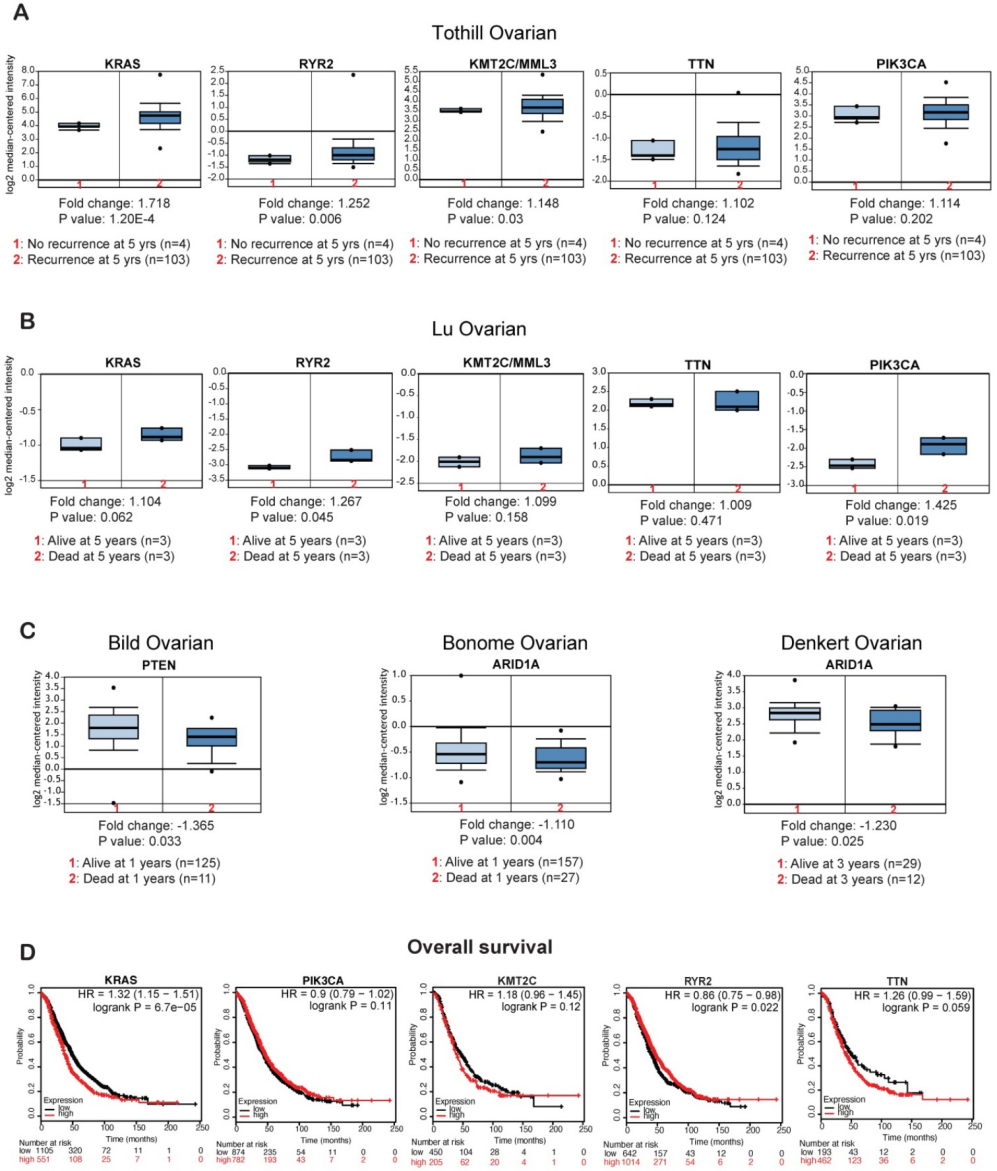
** Effects of overexpressed or repressed genes on the survival of patients with ovarian cancer.** (**A**) The Tothill ovarian dataset from Oncomine analyzed for the effect of the expression levels of the selected genes on disease recurrence at 5 years. (**B**) The Lu ovarian dataset from Oncomine analyzed for the effect of the expression levels of the selected genes on survival at 3 years. (**C**) The Bild ovarian dataset from Oncomine analyzed for the effects of *PTEN* and *ARID1A* expression on survival at 1 year. The Denkert ovarian dataset from Oncomine analyzed for the effect of *ARID1A* expression on survival at 3 years. (**D**) The effect of upregulated genes on overall survival (OS) of patients with ovarian cancer as measured by KM plotter.

Finally, to determine if the genes that display altered mRNA expression levels in ovarian cancer patient samples (*PIK3CA, TTN, RYR2, KMT2C, KRAS, PTEN*, and *ARID1A*) have any clinical value, we checked the effect of their expression on recurrence and survival of patients with ovarian cancer. Patients that experienced recurrence within 5 years of initial treatment had higher *KRAS, RYR2*, and *KMT2C* mRNA expression than those that did not experience recurrence within 5 years (**Figure 6A**). Additionally, patients that died within 5 years of diagnosis had higher *RYR2* and *PIK3CA* mRNA expression than those that survived for at least 5 or more years (**Figure 6B**). By contrast, patients that died within 1 year of diagnosis had lower *PTEN* and patients that died within 1 and 3 years of diagnosis has lower *ARID1A* mRNA expression (**Figure 6C**). Furthermore, to determine if the overexpressed genes affect overall survival (OS) among patients with ovarian cancer, we employed Kaplan-Meier plotter and assessed the effect of the expression levels of these genes on OS of ovarian cancer patients. Increase in *KRAS* expression was associated with significant low OS (**Figure 6D**). However, we also found that increase in* PIK3CA* and *KMT2C* expression were also associated with low OS but not significant (**Table 2; Figure 6D, Supplementary Figure S1A**). In contrast high *PTEN* expression significantly correlated with high OS (**Supplementary Figure S1B**). These results indicate that a subset of the identified genes function as oncogenes and tumor suppressor gene to regulate tumor growth and recurrence and affect the survival of the ovarian cancer patients. In conclusion our study indicates that these identified genes may be useful as biomarkers to predict disease progression, treatment outcomes and overall survival of the patients.

## Discussion

Over the last decade there has been significant advancement in large-scale sequencing technologies, which has revolutionized the clinical arena and immensely affected patient care 38. High-throughput genomic analyses of individual tumors have led to the identification of important cancer targets and the development of personalized therapies 39. Such analyses have also helped to identify novel biomarkers that can predict, diagnose, and monitor various cancers 40, 41.

In our previous studies, researchers have investigated the mutational landscape across major cancer types and identified genetic changes that are common across multiple cancer types and also changes that are specific to certain cancers 42. Here, we performed a detailed analysis of the gynecological malignancies in order to sketch their mutational landscape.

Our results provide information on the prevalence of the different gynecological malignancies, racial/ethnic disparities in the incidence of the gynecological malignancies, and differences in the age at which the malignancies are usually detected. Race based cancer disparities reflects the key role that the genetic differences between different race plays in regulating cancer growth, its aggressiveness and treatment outcome. Diagnosis age affect patient survival and therefore diagnosis of endometrial cancer and ovarian cancer at the later age makes it hard to effectively treat, leading to high mortality rates 43.

Our results show that most of the gynecological malignancies had a high mutational burden, which has been shown to effect cancer-cell growth and progression, treatment outcomes, and patient survival 44. Further, our systematic analysis of several TCGA datasets for uterine, ovarian, cervical, and vaginal cancer revealed 16 important genes that are altered at frequencies >10% in those diseases. We also observed that among 16 identified most altered candidate genes, some of them are shown to functions as either putative driver or have unknown significance (**Supplementary Figure S1C**) in gynecological malignancies. Among the identified genes, *TP53*, *PIK3CA*, *PTEN* and *KRAS* were previously shown to drive other cancers. For example, *TP53* is a known transcription factor that affects the cell cycle and apoptosis in cancer cells 45. Additionally, *PIK3CA* and *PTEN* are known regulators of PI3/AKT signaling, which is a key regulatory pathway that can promote cancer growth and proliferation 46. *KRAS* is a one of the most frequently mutated oncogenes in cancer and has been shown to regulate signaling in several components of the tumor cells. Studies have shown that *KRAS* regulated signaling pathways range from ECM changes to endothelial cell signals or the modulation of cancer-associated fibroblasts and inflammatory/immune cells. These changes promote metabolic, proliferative, migratory, or differentiation ability of cancer cells resulting in increase of their growth, invasion and migration potential 47. We also found that some of the identified genes, including *TTN, MUC16, CSDM3, RYR2, USH2A*, and *SYNE1*, are only altered in gynecological malignancies and not in other cancer types, highlighting their specific role in driving gynecological malignancies. *TTN* mutations have been frequently reported in solid tumors and have been shown to be associated as a predictor of improved outcomes in response to immune checkpoint blockade (ICB) immunotherapy 48. *MUC16* has been identified as a tumor biomarker and serves as novel target for cancer therapy 49. *CSDM3* was also found to be frequently mutated in lung cancer samples, with no clear function 50. *RYR2* is a member of the RyR gene family and has been to be associated with several cancers like melanoma, breast, lymphoma etc. It has been also been established that *RYR2* functionally regulates Ca^2+^ release from sarcoplasmic reticulum into the cytosol and Ca^2+^ levels effects transcription, vesicle secretion, cell proliferation and apoptosis. In tumor cells alteration of *RYR2* regulated intracellular Ca^2+^ levels promotes angiogenesis and cellular migration thus confirming its critical role in tumor growth and progression 51. Mutation in *USH2A* gene is observed in patients with Usher syndrome type II or non-syndromic retinitis pigmentosa, however its function is yet unknown in cancer 52. *SYNE1* gene is shown to be involved in nuclear organization and structural integrity, function of the Golgi apparatus, and cytokinesis. An isoform encoded by *SYNE1* has been reported to be downregulated in ovarian and other cancers, however its function in cancer is not clearly established 53. Thus, in sum based on previous study, role of *CSDM3, USH2A* and* SYNE1* is still not known in any cancer. In addition to that, the function of *TTN, CSDM3, RYR2, USH2A* and *SYNE1* has never been established in ovarian cancer.

**Figure 7:**
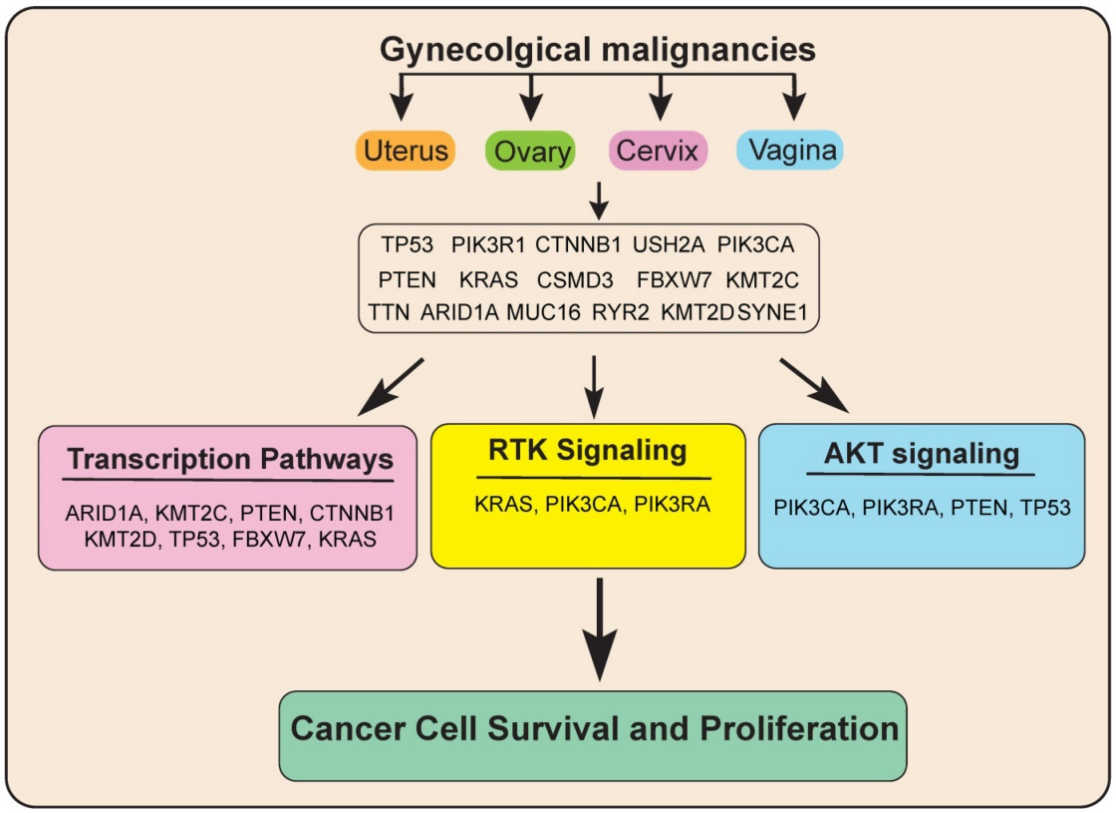
Model showing the important common genetic alterations/signaling pathways that affect the growth and progression of gynecological malignancies.

Next, to know more about the functions of these genes and significantly altered pathway in gynecological malignancies, we performed pathways analysis using these 16 genes via reactome analysis tool. Our results showed that these 16 candidate genes regulate various signaling pathways, including transcription pathways, the AKT signaling pathway, and the Receptor tyrosine kinase (RTK) signaling pathway. Alterations in all these signaling pathways lead to deregulated gene expression leading to uncontrolled cancer growth and proliferation (**Figure 7**).

We also investigated the clinical impact of the identified gene that were either overexpressed or repressed on the recurrence and survival of the ovarian cancer patients. We found that *KRAS, RYR2*, and *KMT2C* overexpression was associated with recurrence after 5 years, and *RYR2* and *PIK3CA* overexpression was associated with mortality within 1 year. By contrast, *PTEN* repression was associated with dead at 1 year and *ARID1A* repression was associated with dead at 1 and 3 years. Additionally, higher expression of *KRAS* was also significantly associated with lower OS of ovarian cancer patients. Previous studies have confirmed the roles of *PIK3CA* and *KRAS* activation and *PTEN* and *ARID1A* inactivation in ovarian cancer 54, 55.

In conclusion, apart from the known regulators, our study identified new regulators of gynecological malignancies that have not been previously reported, including *TTN, KMT2D, USH2A, RYR2*, and *KMT2C*. In future functional studies are needed to understand the specific roles of these genes and the mechanisms by which these genes promote the growth and proliferation of gynecological malignancies and use them for personalized biomarker driven based treatment of gynecological malignancies.

## Materials and Methods

### TCGA dataset analysis using cBioPortal

The cBioPortal for Cancer Genomics website (http://www.cbioportal.org) was used to access the TCGA mRNA expression data. All of the listed TCGA datasets for cervical cancer (cervical squamous cell carcinoma), ovarian/fallopian tube cancer (serous ovarian cancer and small cell carcinoma of the ovary), uterine cancer (endometrial carcinoma, uterine carcinosarcoma/uterine malignant mixed Mullerian tumor, uterine clear cell carcinoma), and vulvar/vaginal cancer (squamous cell carcinoma of the vulva/vagina) were used for the analysis of sample type, diagnosis age, OS in months, mutational burden, and fraction of genomes altered. The TCGA contains 607 samples of cervical cancer, 1,672 samples of ovarian/fallopian tube cancer, 1,799 samples of uterine cancer, and 15 samples of vulvar/vaginal cancer. Mutations and copy-number alterations were analyzed. An oncoprint of each identified gene is shown for different cancer types from various studies including TCGA provisional, TCGA nature, PanCancer Atlas, and others, reflecting genetic alterations such as missense mutations, truncation mutations, in-frame mutations, and other alterations. For ovarian cancer, mRNA expression z-scores (RNA Seq V2 RSEM) were shown for the genes selected using the TCGA provisional data for ovarian cancer. Upregulation and downregulation at the mRNA level, as well as no change in mRNA expression, were shown for the patient samples. For the analysis of missense mutations, truncations, in-frame mutations, and other mutations as well as copy number alteration for all cancer types, the PanCancer Studies containing MSK-IMPACT clinical sequencing cohort, which contains 10,945 samples encompassing many different cancer types, was used 30.

### Oncomine dataset analysis for the mRNA expression of the genes that are overexpressed/repressed transcriptionally and altered with >10% frequency in ovarian cancers

The TCGA ovarian cancer dataset was downloaded from Oncomine (https://www.oncomine.org) and analyzed to determine the expression of the selected genes. The dataset contains genome-wide expression determined using an Affymetrix HGU133A array for a total of 594 samples, including 8 normal ovary samples and 586 ovarian serous cystadenocarcinoma samples. The relative fold-change in gene expression between the cancer samples and the normal samples and its significance are shown in the images download. The Tothill ovarian dataset was used to plot the effect of gene overexpression on disease recurrence 56 The Lu ovarian 57 Bild ovarian 58 Bonome ovarian 59 and Denkert ovarian 60 datasets were used to plot the numbers of surviving and deceased patients.

### The Kaplan-Meier plotter

The prognostic value of the mRNA expression of the mutated genes in ovarian cancer was estimated using the Kaplan-Meier plotter (KM plotter), which is an online database that integrates mRNA expression and clinical data 61. In the database, information on the OS of patients with ovarian cancer (n=1,657) is available. In order to assess the prognostic value of each gene, the patient samples were divided into two cohorts according to the median expression level of the gene of interest. The mRNA expression of a gene above or below the median separates the cases into high expression and low expression. Hazard ratio (HR) is the ratio of the hazard rates corresponding to the conditions described by two levels of an explanatory variable in survival analysis. HR ratio, 95% confidence intervals and log rank P are presented on the main plots. We analyzed the correlations between the mRNA levels and the OS of the patients in the ovarian cancer datasets. Briefly, the selected genes (*KRAS, TTN, PIK3CA, KMT2C*, and *RYR2*) were uploaded into the database to obtain the Kaplan-Meier OS plots. HR ratio with 95% confidence intervals and log-rank *p*-values were estimated as shown on the https://kmplot.com/analysis/index.php?p=service&cancer=ovar webpage. To minimize the false discovery rate, we considered *p*<0.05 as a minimum edge and to be to be statistically significant. The chosen Affymetrix ID of each specific gene identified as a driver of ovarian cancer is listed in Table 2.

## Supplementary Material

Supplementary Figure S1.

## Figures and Tables

**Table 1:** Gynecological cancer type and subtype and the study source: TCGA datasets from cBioportal

Cancer type	Cancer subtype	Study source
Cervical cancer	Cervical squamous cell carcinoma	TCGA, PanCancer Atlas
	Cervical squamous cell carcinoma and Endocervical adenocarcinoma	TCGA, Provisional
Ovarian/fallopian tube Cancer	Ovarian serous cystadenocarcinoma	TCGA, Nature 2011
	Ovarian serous cystadenocarcinoma	TCGA, PanCancer Atlas
	Ovarian serous cystadenocarcinoma	TCGA, Provisional
	Small cell carcinoma in the ovary	MSKCC, Nat Genet 2014
Uterine cancer	Endometrial cancer	MSK, 2018
	Uterine carcinosarcoma	John Hopkins, Nat Commun 2014
	Uterine carcinosarcoma	TCGA, PanCancer Atlas
	Uterine carcinosarcoma	TCGA, Provisional
	Uterine corpus endometrial carcinoma	TCGA, Nature 2013
	Uterine corpus endometrial carcinoma	TCGA, Provisional
	Uterine corpus endometrial carcinoma	TCGA, PanCancer Atlas
	Uterine clear cell carcinoma	NIH, Cancer 2017
Vulvar/vaginal cancer	Squamous cell carcinoma of vulva	CUK, Exp Mol Med 2018

**Table 2:** The KM Plotter Affymetrix ID of each specific gene in ovarian cancer

Gene	Affymetrix ID
KRAS	214352_s_at
PIK3CA	204369_at
KMT2C	1557158_s_at
RYR2	2077557_s_at
TTN	241791_at

## Abbreviations

TCGA: The Cancer Genome Atlas
TP53: tumor protein 53
PIK3CA: phosphatidylinositol-4,5-bisphosphate 3-kinase, catalytic subunit alpha
PTEN: phosphatase and tensin homolog
TTN: Titin
ARID1A**: AT-rich interactive domain-containing protein 1A
MUC16: mucin 16
PIK3R1: phosphatidylinositol 3-kinase regulatory subunit alpha
KMT2D: histone-lysine N-methyltransferase 2D
CSMD3: CUB and sushi multiple domains 3
RYR2: ryanodine receptor 2
CTNNB1: catenin beta-1
FBXW7: F-box/WD repeat-containing protein 7
USH2A: usherin
KMT2C: lysine N-methyltransferase 2C
SYNE1: spectrin repeat-containing nuclear envelope protein 1
