# Supplementary material for: Identification of the Mutational Landscape of Gynecological Malignancies
Source: J Cancer. 2020 Jun 8;11(16):4870–83. doi: 10.7150/jca.46174 (PMC7330690; doi:10.7150/jca.46174)
Supplement: Supplementary file 1 — Supplementary Figure S1. [file jcav11p4870s1.pdf]

Supplementary Figure legend

Supplementary Figure S1. Effect of TTN, PTEN and ARID1A expression on Overall survival of patients with gynecological malignancies using TCGA dataset from cBioportal and Kaplan-Meier (KM) plotter respectively.

(**A**) The effect of TTN expression on the overall survival in months of patients with ovarian/fallopian tube cancer, cervical cancer, and uterine cancer in the TCGA dataset from cBioportal. (**B**) The effect of downregulated regulated genes (PTEN and ARID1A) on overall survival (OS) of patients with ovarian cancer as measured by KM plotter. (**C**) Analysis of TCGA dataset from cBioportal for different kinds of gynecological malignancies to identify the genes that either carry driver mutations or random mutations/mutation with unknown significance.

Supplementary Figure S1

A

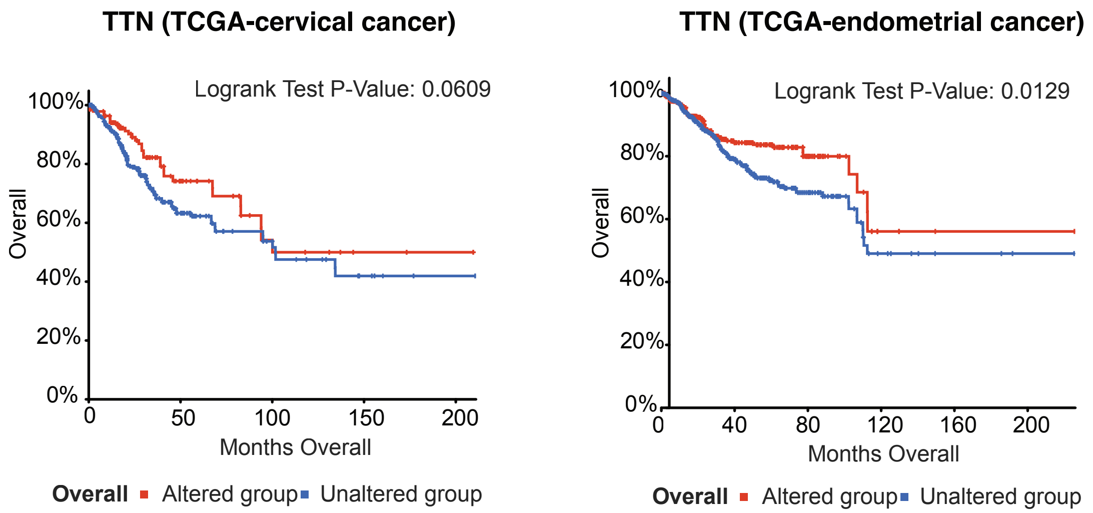

B

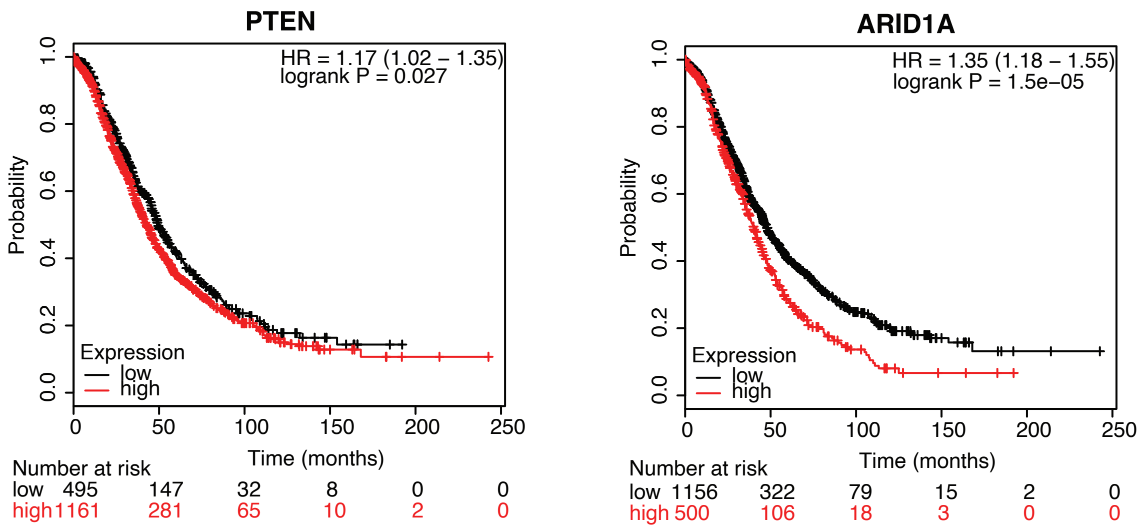

C

Gene	Driver/random mutation status
TP53	Putative driver
PIK3CA	Putative driver
PTEN	Putative driver
TTN	Unknown significance and will require functional validation
ARID1A	Putative driver
MUC16	Unknown significance and will require functional validation
PIK3R1	Putative driver
KMT2D	Putative driver
CSMD3	Unknown significance and will require functional validation
RYR2	Unknown significance and will require functional validation
CTNNB1	Putative driver
FBXW7	Putative driver
USH2A	Unknown significance and will require functional validation
KMT2C	Putative driver
SYNE1	Unknown significance and will require functional validation
KRAS	Putative driver
